# Green tea catechins: protectors or threats to DNA? A review of their antigenotoxic and genotoxic effects

**DOI:** 10.1007/s00204-025-04063-7

**Published:** 2025-05-13

**Authors:** María del Carmen García-Rodríguez, Sam Kacew

**Affiliations:** 1 Laboratorio de Antimutagénesis, Anticarcinogénesis y Antiteratogénesis Ambiental, Facultad de Estudios Superiores—Zaragoza, Universidad Nacional Autónoma de México (UNAM), Mexico City, Mexico; 2 McLaughlin Centre for Population Health Risk Assessment, University of Ottawa, Ottawa, ON Canada

**Keywords:** Reactive oxygen species, Endogenous antioxidant systems, DNA repair, Apoptosis, *Camellia sinensis*, Natural compounds

## Abstract

This review examines the dual behavior of green tea catechins (GTCs), demonstrating the compound’s ability to protect against oxidative stress and DNA damage while also potentially inducing genotoxicity under certain conditions. This duality may be attributed to their capacity both to scavenge free radicals and to generate these species via autooxidation. GTCs’ antigenotoxic activities are mediated by multiple mechanisms, including reactive oxygen species (ROS) scavenging, regulation of endogenous antioxidant system (EAS), DNA repair, selective apoptosis of genetically compromised cells, epigenetic modulation, and metal ion (Cu, Fe, Zn) chelation—all of which collectively maintain cellular homeostasis and help reduce inflammation. However, at specific concentrations and in certain cellular conditions, GTCs’ prooxidant effects—i.e., high ROS levels—might damage DNA and promote pro-apoptotic processes, potentially benefiting elimination of malignant cells. In contrast, lower ROS levels might stimulate antioxidant defenses via Nrf2 activation. Although evidence from both in vitro and in vivo studies indicates that GTCs consumption offers significant protection against diseases linked to oxidative DNA injury, the prooxidant properties of GTCs warrant careful consideration. Future research might focus on (1) optimizing GTC formulations for improved bioavailability, (2) assessing long-term outcomes, (3) evaluating toxicity at higher doses, and (4) investigating gut microbiome interactions. The dual antigenotoxic and genotoxic actions of GTCs indicate the potential role in preventive and complementary medicine, aligning with sustainable beneficial health strategies utilizing natural compounds.

## Introduction

Green tea catechins (GTCs) have attracted considerable attention due to antioxidant properties and potential chemopreventive effects (Musial et al. 2020; Samanta 2022). However, a closer examination indicates that these compounds might also exhibit prooxidant activity, which under certain conditions leads to genotoxic damage (García-Rodríguez et al. 2022). Although several investigators reported the antigenotoxic efficacy of GTCs against heavy metals, radiation, and other stressors (García-Rodríguez et al. 2022, 2023; Ouyang et al. 2020), the conditions that drive a transition from antigenotoxic to genotoxic behavior remain only partially understood. Further, published contrasting findings regarding GTCs antioxidant yet simultaneously prooxidant potential often stemming from variations in experimental design (Farhan 2022; Furukawa et al. 2003; García-Rodríguez et al. 2022) add complexity to the current understanding. Because genotoxicity, oxidative stress, and various chronic degenerative diseases (including cancer) are closely interconnected (Farhan 2022; Jomova et al. 2023), there has been growing interest in green tea’s antioxidant components for prevention and adjunctive therapy in conditions involving DNA damage (García-Rodríguez et al. 2023; Ghosh et al. 2024; Samanta 2022). Despite evidence that GTCs may alleviate oxidative stress and reduce genotoxic injury (García-Rodríguez et al. 2022, 2023; Mokra et al. 2023), it is essential to acknowledge that these compounds might also induce oxidative stress, particularly via autooxidation ultimately leading to genotoxic outcomes (Ouyang et al. 2020; Udroiu et al. 2019). Taking into account the antioxidant, prooxidant properties of GTCs, this review sought to examine both the antigenotoxic and genotoxic effects attributed to GTC exposure, with emphasis on the molecular mechanisms involved and implications for human health.

## Green tea catechins (GTCs)

GTCs are prominent polyphenols found in green tea, a beverage derived from the leaves of *Camellia sinensis* and consumed globally, often surpassing coffee, beer, wine, and soft drinks in popularity in certain countries (Cabrera et al. 2006). Historically, green tea was the first variety of tea discovered and remains unique as a non-fermented type (Zhao et al. 2022). Cultivation primarily occurs in tropical and temperate regions of Asia such as China, India, Sri Lanka, and Japan and extends to parts of Africa and South America. The leaves of *Camellia sinensis* are processed into various tea types (green, black, oolong, and white), each defined by distinct processing methods. Notably, the steaming of leaves in green tea production inactivates polyphenol oxidase, thereby preserving catechins, the most abundant polyphenol that imparts a characteristic flavor and health benefits (Samanta 2022). In contrast, black tea undergoes full oxidation while oolong tea is partially oxidized, leading to unique flavors and properties (Samanta 2022; Senanayake 2013).

### Structural and functional properties

**Fig. 1:**
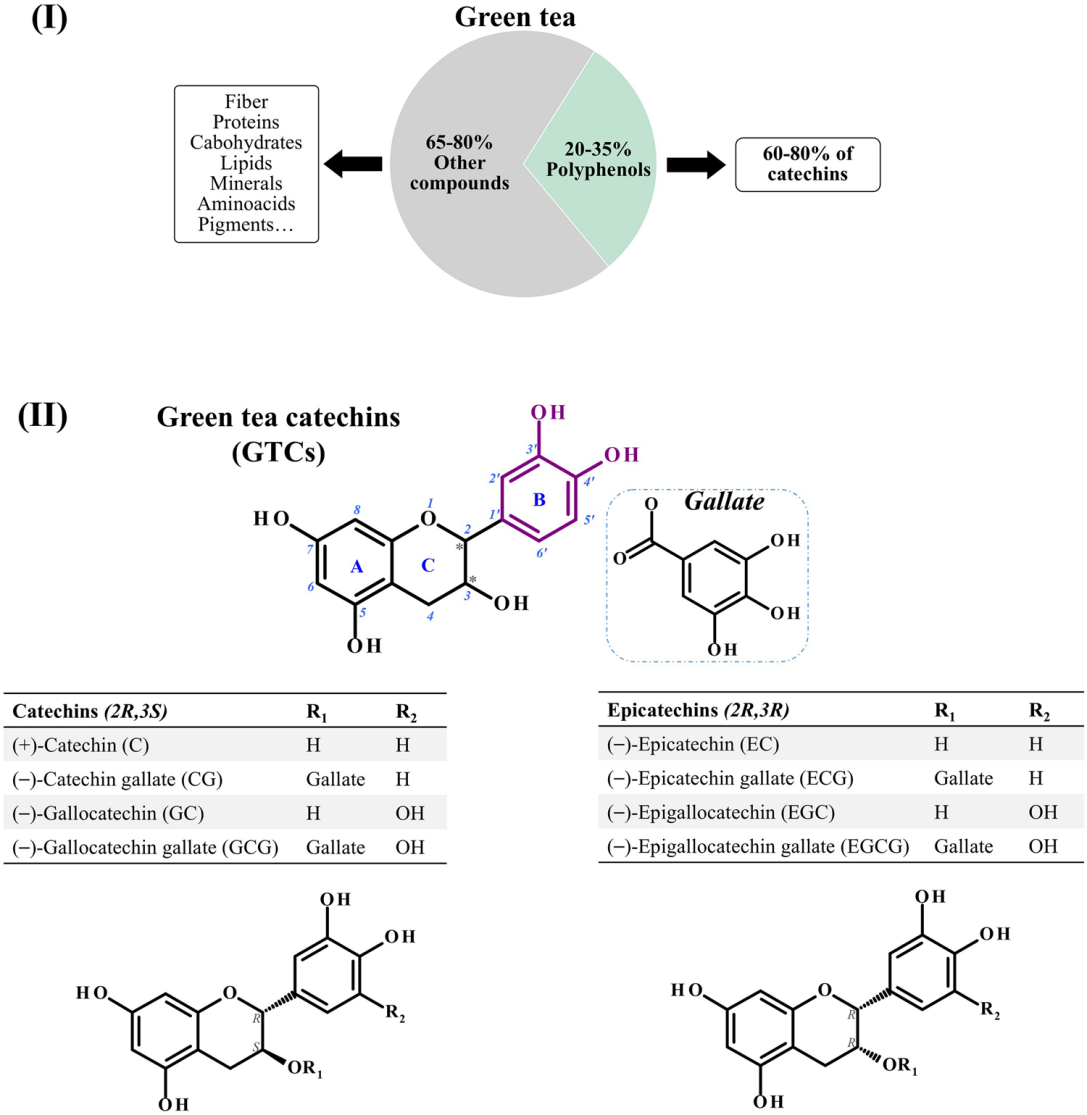
(I) Composition of green tea, emphasizing the proportion of catechins. (II) Chemical structures of GTCs in their (*2R,3S*) and *epi* forms with a (*2R,3R*) configurations. (III) Structural overview of GTCs, showing two aromatic rings (**A**, **B**) connected by a three-carbon bridge (**C**). A catechol moiety is present on ring B, hydroxyl (OH) groups occupy the 5- and 7-positions of ring A, and a gallate group attaches at the 3-position of ring C. The inset details the gallate group’s structure

Polyphenols constitute approximately 20–35% of green tea dry weight (Zhang et al. 2019). GTCs account for 60–80% of the polyphenols (Fig. 1-I), with (−)-epigallocatechin-3-gallate (EGCG) being the most abundant encompassing approximately 10% of the dry weight in fresh buds and young leaves (Samanta 2022). The primary GTCs include (+)-catechin (C), (−)-catechin gallate (CG), (−)-gallocatechin (GC), (−)-epicatechin (EC), (−)-epigallocatechin (EGC), (−)-epicatechin-3-gallate (ECG), (−)-gallocatechin gallate (GCG), and EGCG. Structurally, GTCs feature two benzene rings (A- and B-rings) connected by a dihydropyran heterocycle (the C-ring), which contains a hydroxyl (OH) group at carbon 3. The A-ring resembles a resorcinol moiety, while the B-ring resembles a catechol moiety. Two chiral centers at carbons 2 and 3 enable classification into two stereochemical groups: compounds with a (*2R,3S*) configuration—C, CG, GC, and GCG—and their *epi* forms with a (*2R,3R*)—EC, EGC, ECG, and EGCG (Fig. 1-II). These structural distinctions influence biologic activity and determine how GTCs interact with various cellular targets (Yang et al. 2018b).

## Antigenotoxic effects

Genotoxic damage arises when a chemical disrupts DNA or other cellular components, compromising genomic integrity and potentially leading to diseases such as cancer (Eastmond et al. 2009). Consequently, identifying compounds that either induce or protect against genotoxic damage is of critical importance. Both in vitro and in vivo investigations indicated that GTCs may reduce genotoxic effects induced by carcinogens, heavy metals, tobacco compounds, radiation, and medical conditions such as type 1 diabetes, bladder dysfunction, myocardial injury, hepatic ischemia–reperfusion, and alcoholic liver disease (Table 1). In particular, green tea extracts or infusions were reported to diminish genotoxic damage by limiting DNA fragmentation and altering DNA content following exposure to arsenic (As), iron (Fe), chromium (Cr), cyclophosphamide, patulin, and in conditions such as diabetic nephropathy (Acharyya et al. 2015; Al-Basher 2019; García-Rodríguez et al. 2013; Ladeira et al. 2021; Zanchi et al. 2015). In addition, matcha green tea and specific GTCs formulations (P60-GT) exhibited comparable protective benefits against radiation, Cr, and patulin (Hamed et al. 2025; Hernández-Cortés et al. 2025; Song et al. 2014). Notably, individual catechins particularly EGCG—were shown to be effective in reducing As- or Cr-induced genotoxicity by preventing double-strand DNA breaks (García-Rodríguez et al. 2014, 2016, 2021; Kaushal et al. 2019). A dose–response pattern often emerges, wherein pretreatment with EGCG or P60-GT yielding significant decreases in micronuclei (MN) frequency, chromosomal aberrations (CA), DNA content alterations, and comet tail length compared to post-treatment (Hernández-Cortés et al. 2025; Kaushal et al. 2019; Song et al. 2014; Xie et al. 2020). Further, in vitro observations demonstrated that EGCG markedly lowered number of DNA double-strand breaks induced by carcinogens and radiation (Taghavi et al. 2020; Wang et al. 2022; Xie et al. 2020). Similarly, green tea extract was found to diminish mercury (Hg)-induced number of MN in *Allium cepa* L. (Çavuşoğlu et al. 2022).

**Table 1:** Overview of in vitro and in vivo studies demonstrating antigenotoxic effects of green tea catechins

Treatment **(**duration)	Study design	Experimental model	Key observations/findings	Bioactivities demonstrated and *target condition*	References
Green tea infusion 100 mg/kg (42 days by gavage)	In vivo	Male Wistar rats	⇩DNA-damaged cells ⇧ GST ⇩ Apoptotic cell	Antigenotoxic Antioxidant Anti-apoptotic Renoprotective*—Type 1 diabetes*	Ladeira et al. (2021)
Green tea infusion 250 mg/kg (14 days orally)	In vivo	Male Swiss albino mice	⇩DNA breaks ⇧ GPx and GST	Antigenotoxic Antioxidant Reproprotective*—Reproductive system* *(Cyclophosmamide)*	Zanchi et al. (2015)
Matcha green tea 150 mg/kg (14 days orally)	In vivo	Female Wistar albino rats	⇩DNA fragmentation ⇧ SOD and GST ⇩ NF-κB/MAPK signaling	Antigenotoxic Antioxidant Anti-inflammatory Hepatoprotective*—Liver damage* *(Ionizing radiation)*	Hamed et al. (2025)
Green tea extract 10 mg/kg (28 days by gavage)	In vivo	Female albino rats	⇩DNA fragmentation ⇧ SOD and CAT	Antigenotoxic Antioxidant Hepatoprotective*—Metal toxicity (As)*	Acharyya et al. (2015)
Green tea extract 100 mg/kg (60 days orally)	In vivo	Male albino Sprague–Dawley rats	⇧DNA content ⇧ TAC ⇩ Apoptotic cell ⇩ cyt c	Antigenotoxic Antioxidant Anti-apoptotic Hepatoprotective*—Molecular fibrogenesis* *(Fe)*	Al-Basher (2019)
Green tea extract 4 mg/day (14 days orally)	In vivo	Female CBA/Ca mice	⇩DNA hypomethylation ⇧ DNA methyltransferase ⇧ GSH	Antigenotoxic Antioxidant Chemopreventive*—DMBA*	Szabo et al. (2021)
Green tea extracts 30 mg/kg (Single by gavage)	In vivo	Male CD-1 (ICR) mice	⇩Micronuclei ⇧ Apoptotic cell ⇩ Necrotic cell	Antigenotoxic Pro-apoptotic Cytoprotective*—Metal toxicity (Cr)*	García-Rodríguez et al. (2013)
P60-GT 25, 50, 100 mg/kg (7 days by ip)	In vivo	Male Kunming mice	⇩Micronuclei ⇩ Chromosomal aberrations ⇧ GSH ⇩ ROS ⇩ cas-3 ⇩ BAX ⇧ Viable cells	Antigenotoxic Antioxidant Cytoprotective Hepatoprotective*—Liver damage* *(Patulin)*	Song et al. (2014)
P60-GT 15, 30, 45 mg/kg (Single by gavage)	In vivo	Male Hsd:ICR mice	⇩Micronuclei ⇧ 8-OHdG ⇩ Apoptotic cell [15 mg/kg] ⇧ Apoptotic cell [30, 45 mg/kg] ⇩ Necrotic cell	Antigenotoxic Anti-apoptotic Pro-apoptotic Cytoprotective*—Metal toxicity (Cr)*	Hernández-Cortés et al. (2025)
Green tea polyphenols, EC, EGCG 20 mg/kg (Single by gavage)	In vivo	Female and male Sprague Dawley rats	⇩N7-GA- Gua ⇩ N3-GA-Ade	Antigenotoxic Protection oxidative DNA damage Chemopreventive*—Acrylamide*	Zhang et al. (2022)
EGCG 10 mg/kg (Single by ip)	In vivo	Male CD-1 (ICR) mice	⇩Micronuclei	Antigenotoxic Cytoprotective*—Metal toxicity (Cr)*	García-Rodríguez et al. (2014)
EGCG 10 mg/kg (Single by gavage)	In vivo	Male Hsd:ICR mice	⇩Micronuclei ⇧ Apoptotic cell ⇧ Cell viability	Antigenotoxic Pro-apoptotic Cytoprotective*—Metal toxicity (Cr)*	García-Rodríguez et al. (2016)
EGCG 8.5 mg/kg (Single by gavage)	In vivo	Male Hsd:ICR mice	⇩Micronuclei ⇩ 8-OHdG ⇧ TAC ⇩ GSH ⇧ SOD ⇩ Apoptotic cell ⇧ Cell viability	Antigenotoxic Protection oxidative DNA damage Antioxidant Anti-apoptotic Cytoprotective*—Metal toxicity (Cr)*	García-Rodríguez et al. (2021)
EGCG 25, 50 mg/kg) (15 days orally)	In vivo	Female balb/C mice	⇩DNA fragmentation ⇩ % DNA and tail length ⇩ Micronuclei ⇩ Chromosomal aberrations ⇧ CAT, SOD, GST and GR ⇧ GSH ⇩ ROS	Antigenotoxic Antioxidant Hepatoprotective Renoprotective*—Metal toxicity (As)*	Kaushal et al. (2019)
EGCG 12.5, 25 mg/kg (5 days by ip)	In vivo	Male C57BL/6 J mice	⇩DNA double-strand breaks ⇩ 8-OHdG ⇧ Nrf-2 ⇧ GPx, HO-1 ⇩ ROS ⇩ Apoptotic cells	Antigenotoxic Protection oxidative DNA damage Antioxidant Anti-apoptotic Radioprotective*—Ionizing radiation*	Xie et al. (2020)
Green tea extract 190, 380 mg/L (40 min)	In vitro	*Allium cepa L*	⇩Micronuclei ⇩ Chromosomal aberrations ⇩ ROS ⇩ SOD, CAT ⇩ MDA	Antigenotoxic Antioxidant Cytoprotective*—Metal toxicity (Hg)*	Çavuşoğlu et al. (2022)
EGCG 2 μM (30 min)	In vitro	HIEC	⇩DNA double-strand breaks ⇩ 8-OHdG ⇩ γH2AX ⇧ GPx, HO-1 ⇧ Nrf-2 ⇩ ROS ⇩ Apoptotic cells	Antigenotoxic Protection oxidative DNA damage Antioxidant Anti-apoptotic Radioprotective*—Ionizing radiation*	Xie et al. (2020)
EC, EGCG 12,5, 25 y 50 μg/mL (2 h)	In vitro	HepG2 cells	⇩DNA breaks ⇧ DNA repair PARP ⇩ Cleaved PARP ⇩ Bax ⇩ cas-3	Antigenotoxic Anti-apoptotic Cytoprotective Hepatoprotective*—Acrylamide and glycidamide*	Wang et al. (2022)
EGCG 20, 50 μM (24 h)	In vitro	Human lymphocytes	⇩Micronuclei ⇧ GSH ⇩ MDA	Antigenotoxic Antioxidant*—Malathion*	Taghavi et al. (2020)
Green tea polyphenols 200 mg/L (24 h)	In vitro	IPEC-J2 cells	⇧Transcription levels T-SOD, CAT, GSH-Px ⇧ T-SOD, CAT, GSH-Px ⇩ ROS ⇩ Apoptotic cells ⇧ Cell viability	Antioxidant Anti-apoptotic Cytoprotective*—Fluoride*	Xie et al. (2025)
EGCG 10 μM (30 min)	In vitro	Adult human ventricular cardiomyocytes (AC16)	⇧TAC ⇧ SOD and CAT ⇩ ROS ⇧ Bcl-2 ⇩ Bax ⇩ Apoptotic cells ⇩ IL-8	Antioxidant Anti-apoptotic Anti-inflammatory Cardioprotective*—Cigarette smoke*	Liang et al. (2019)

⇩ Decrease or inhibition; ⇧ Increase

### Free radical scavenging and DNA protection

**Fig. 2:**
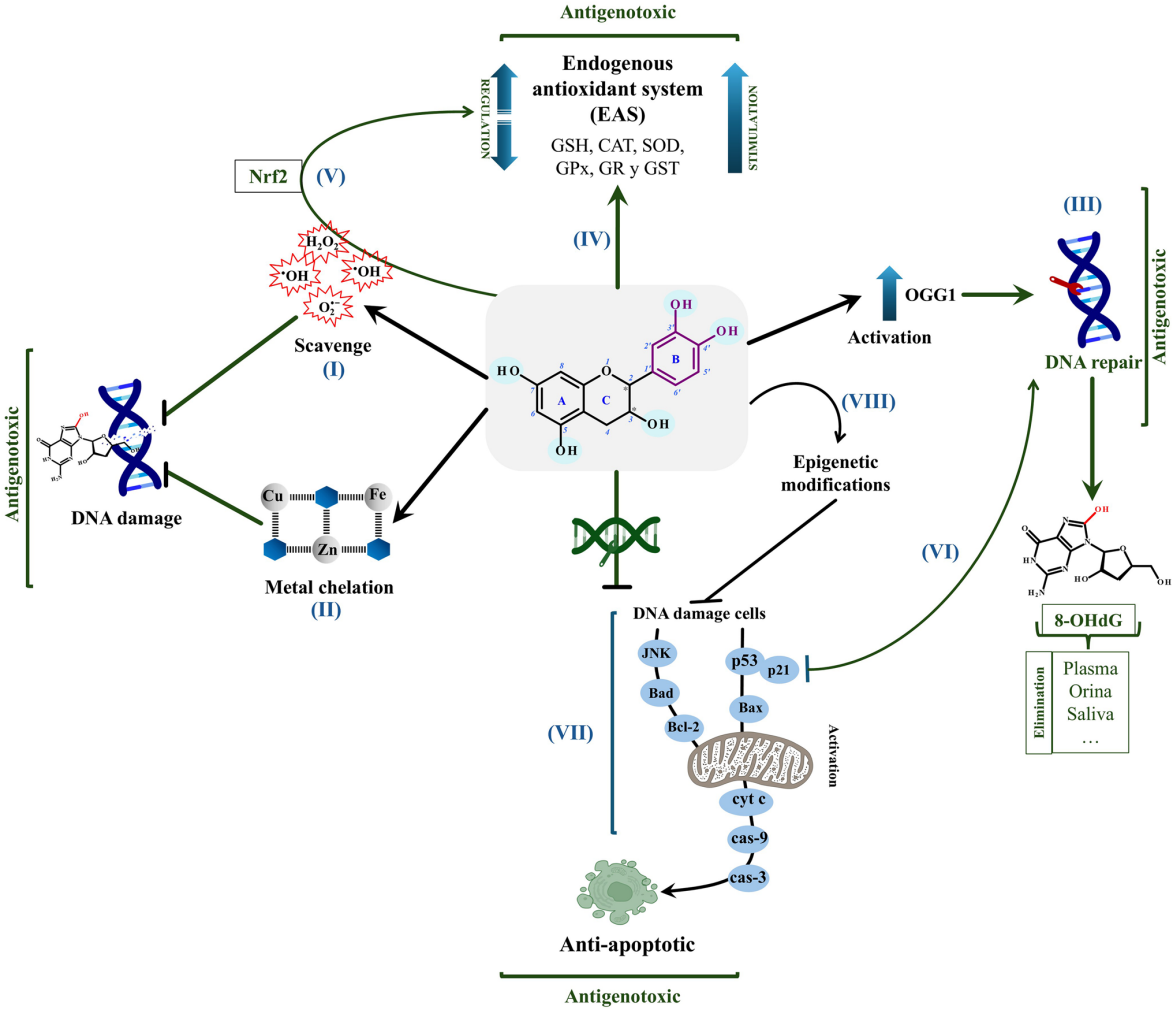
Mechanistic overview of green tea catechins’ antigenotoxic effects: (I) ROS neutralization–GTCs effectively scavenge reactive oxygen species (ROS) via their hydroxyl (OH) groups, preventing oxidative DNA damage. through their hydroxyl (OH) groups, thereby preventing oxidative DNA damage. (II) Metal chelation–GTCs bind metal ions (Cu, Fe, Zn), minimizing oxidative stress and safeguarding cellular components. (III) DNA repair pathway activation–GTCs upregulate essential DNA repair mechanisms, including OGG1, to correct oxidative lesions and maintain genomic stability. (IV) Modulation of the endogenous antioxidant system (EAS)–GTCs enhance total antioxidant capacity (TAC) and regulate key antioxidants (e.g., GSH, CAT, SOD), helping to sustain redox balance and reduce oxidative stress. (V) Nrf2 activation–Nrf2 further augments GTCs’ antigenotoxic effects by fortifying cellular defenses against oxidative damage. (VI) DNA repair facilitation–GTCs mitigate genotoxic injury by downregulating pro-apoptotic proteins involved in cell death signaling. (VII) Apoptosis induction–GTCs initiate apoptosis to eliminate genetically compromised cells when DNA damage cannot be repaired, thus preventing propagation of damaged DNA. (VIII) Epigenetic regulation–GTCs influence gene expression, contributing to the maintenance of genomic integrity and stability

The molecular structure of GTCs particularly their OH groups, might be responsible for the observed defensive effects against genotoxic damage. These OH groups scavenge free radicals by donating hydrogen atoms (Hajam et al. 2023), a key strategy for maintaining genomic stability and preventing DNA strand breaks. In agreement with these observations, Chow and Hakim (2011) reported that smokers consuming 4 cups of decaffeinated green tea daily for 4 months exhibited lower levels of 8-hydroxydeoxyguanosine (8-OHdG), a key biomarker of oxidative stress and genotoxicity (Valavanidis et al. 2009). When hydroxyl radicals (•OH) interact with guanine (2-deoxyguanosine), the resulting radical initiates the formation of 8-OHdG through electron abstraction and keto-enol tautomerism, culminating in 8-oxo-7,8-dihydro-2-deoxyguanosine (8-oxodG) formation. Similarly, both in vivo and in vitro experiments showed fewer double-strand DNA breaks with reduced 8-OHdG levels in human intestinal epithelial cells (HIEC) subjected to ionizing radiation (Xie et al. 2020) and in mice exposed to Cr(VI) (García-Rodríguez et al. 2021). GTCs also inhibit the formation of DNA adducts such as N7-(2-carbamoyl-2-hydroxyethyl)guanine (N7-GA-Gua) and N3-(2-carbamoyl-2-hydroxyethyl)adenine (N3-GA-Ade), both induced by acrylamide exposure (Zhang et al. 2022). These findings suggest that neutralizing •OH might help inhibit genotoxic damage, a protective effect linked to GTCs. Consequently, the structural composition of GTCs is critical in mitigating genotoxic harm through reactive oxygen species (ROS) scavenging and 8-OHdG inhibition (Fig. 2-I).

It is noteworthy that Shi et al. (2000) found that EGCG scavenges •OH at a rate of 4.62 × 10^11^ M^−1^ s^−1^, surpassing well-known antioxidants such as ascorbate, glutathione (GSH), and cysteine. In a 2,2-diphenyl-1-picrylhydrazyl (DPPH) radical-scavenging assay, GTCs effectiveness followed the pattern EGCG > ECG > EGC > EC; a total oxy-radical scavenging capacity (TOSC) assay similarly identifying EGCG and ECG as the most potent antioxidants, followed by EGC, EC, and C (Kang et al. 2010). Further protection from genotoxic damage is closely tied to specific chemical features of GTCs, namely, an ortho-3′,4′-dihydroxyl or a 3′,4′,5′-trihydroxyl group in the B-ring, a gallate group at the 3-position of the C-ring, and OH groups at the 5- and 7-positions on the A-ring (Senanayake 2013). Along with stabilizing free radicals and enhancing radical-scavenging capacity, these structural elements might also enhance GTCs’ metal-chelating capabilities (Aggarwal et al. 2022). Farhan et al. (2017, 2022) found that ortho-hydroxyl groups significantly elevated metal-chelating capacity, especially with Cu(II), by promoting its reduction to Cu(I) in cancer lines. This multifunctional chelation demonstrates the potential of GTCs to protect against oxidative stress and metal-induced DNA damage (Fig. 2-II).

### DNA repair pathways

Although ROS scavenging and inhibition are critical to DNA protection, these mechanisms sometimes prove insufficient, necessitating DNA repair processes as an additional layer of antigenotoxic support. Ho et al. (2014) found that GTCs activate redox-sensitive cytoprotective pathways and promote post-translational modifications of DNA repair enzymes, notably 8-oxoguanine DNA glycosylase 1 (OGG1). Several investigators noted that green tea treatment lowered 8-OHdG adduct levels in the lungs and liver of As-exposed mice while enhancing OGG1 expression (Mizoi et al. 2005; Sinha and Roy 2011). In addition, 8-OHdG repair levels in the plasma were shown to increase in mice pre-treated with P60-GT prior to Cr(VI) exposure (Hernández-Cortés et al. 2025). These findings demonstrate OGG1’s essential role in repair of oxidative DNA damage and suggest that GTCs mitigate genotoxicity by enhancing DNA repair pathways (Fig. 2-III). Consequently, fewer oxidative DNA lesions in tissues along with elevated levels of 8-OHdG in body fluids point to activation of repair mechanisms that eliminate mutagenic lesions initiated by •OH.

### Endogenous antioxidant system (EAS)

Regulating the EAS (Fig. 2-IV) is another key mechanism through which GTCs protect against genotoxic harm. When ROS levels disrupt redox balance, the EAS restores homeostasis by stabilizing or eliminating these reactive species. This system primarily depends upon enzymes such as superoxide dismutase (SOD), which converts superoxide (O_2_•⁻) into oxygen and hydrogen peroxide (H_2_O_2_); subsequently, catalase (CAT) and glutathione peroxidase (GPx) reduce H_2_O_2_ into water and oxygen. GPx, in turn, relies on GSH, which, upon oxidation to glutathione disulfide (GSSG), is regenerated by glutathione reductase (GR) (Jomova et al. 2023). Thus, preserving a balance between ROS production and elimination is key to maintaining cellular redox homeostasis. Investigations in rodent models suggest that green tea extracts and EGCG might decrease genotoxic damage by enhancing EAS capacity, partly via increasing total antioxidant capacity (TAC) (Al-Basher 2019; García-Rodríguez et al. 2021). GTCs exhibit antigenotoxic effects and modulate various antioxidant components, including activities of SOD, GSH, CAT, GPx, GR, and glutathione S-transferase (GST), in xenobiotic-exposed models (Acharyya et al. 2015; Çavuşoğlu et al. 2022; Hamed et al. 2025; Kaushal et al. 2019; Ladeira et al. 2021; Song et al. 2014; Taghavi et al. 2020; Xie et al. 2020; Xie et al. 2025; Zanchi et al. 2015). Further evidence indicates that GTCs-mediated EAS activation correlates with lower ROS levels, specifically H_2_O_2_ and O_2_•⁻ (Çavuşoğlu et al. 2022; Kaushal et al. 2019; Xie et al. 2020, 2025). In addition, GTCs upregulate nuclear factor erythroid 2–related factor 2 (Nrf2), a key regulator of the oxidative stress response, via ROS generation (Na and Surh 2008). Nrf2 controls the transcription of genes essential for redox balance, including glutamate cysteine ligase (GCL), GPx, and heme oxygenase-1 (HO-1). Xie et al (2025) observed in vitro that GTCs enhanced the transcription of antioxidants including total-SOD, CAT, and GSH-Px activities. Further, both in vivo and in vitro studies demonstrated that EGCG increased Nrf2 expression and elevated target antioxidant proteins in bladder and lung tissues, as well as in HIEC (Gu et al. 2018; Shanmugam et al. 2020; Xie et al. 2020). It has been proposed that GTCs trigger Nrf2 activation through ROS formation including singlet oxygen (^1^O_2_), •OH, O_2_•⁻, and primarily H_2_O_2_. Under physiologic conditions (pH 7.4, 37 °C), EGCG might autooxidize to o-quinone through the dehydrogenation of its phenolic OH groups; in the presence of air, intermediates such as O_2_•⁻ and EGCG radicals (•EGCG) form, stimulating oxidative reactions that produce various ROS capable of activating Nrf2 (Akagawa et al. 2003; Ishii et al. 2008; Ouyang et al. 2020). In this manner, Nrf2 promotes antioxidant defenses against oxidative stress and enhance the antigenotoxic effects of GTCs (Fig. 2-V).

### Modulation of apoptosis

In both in vivo and in vitro studies, GTC treatment that reduced genotoxic damage was accompanied by decreased levels of apoptosis (Al-Basher 2019; García-Rodríguez et al. 2021; Hernández-Cortés et al. 2025; Ladeira et al. 2021; Wang et al. 2022; Xie et al. 2020), indicating a link between apoptosis modulation and antigenotoxic properties of GTCs. Song et al. (2014) found that P60-GT treatment diminished pro-apoptotic proteins such as caspase-3 (cas-3) and Bax in mice exposed to patulin. In vitro experiments similarly showed that treatment with EC and EGCG decreased these pro-apoptotic markers while concurrently elevating the expression of the DNA repair protein PARP and lowering levels of cleaved-PARP, Bax and cas-3 in HepG2 cells exposed to acrylamide and glycidamide, as well as in adult human ventricular cardiomyocytes exposed to cigarette smoke (Liang et al. 2019; Wang et al. 2022). In addition, Hernández-Cortés et al. (2025) reported that a 15 mg/kg dose of P60-GT exerted anti-apoptotic effects, whereas higher doses (30 and 45 mg/kg) promoted apoptosis in mice exposed to Cr(VI). This dose-dependent response as evidenced by reduced MN formation and lower numbers of apoptotic cells emphasizing the role of apoptosis in mitigating CrO₃-induced genotoxicity. Thus, the anti-apoptotic effects of GTCs may be attributed to (1) downregulation of pro-apoptotic proteins in conjunction with upregulation of DNA repair proteins, and (2) protection of DNA, which minimizes the number of genetically compromised cells and thereby lowering the apoptotic load. Consequently, GTCs play a critical role in maintaining genomic stability by preventing DNA damage and activating repair pathways mediated by apoptosis (Fig. 2-VI, VII).

## Genotoxic effects

**Fig. 3:**
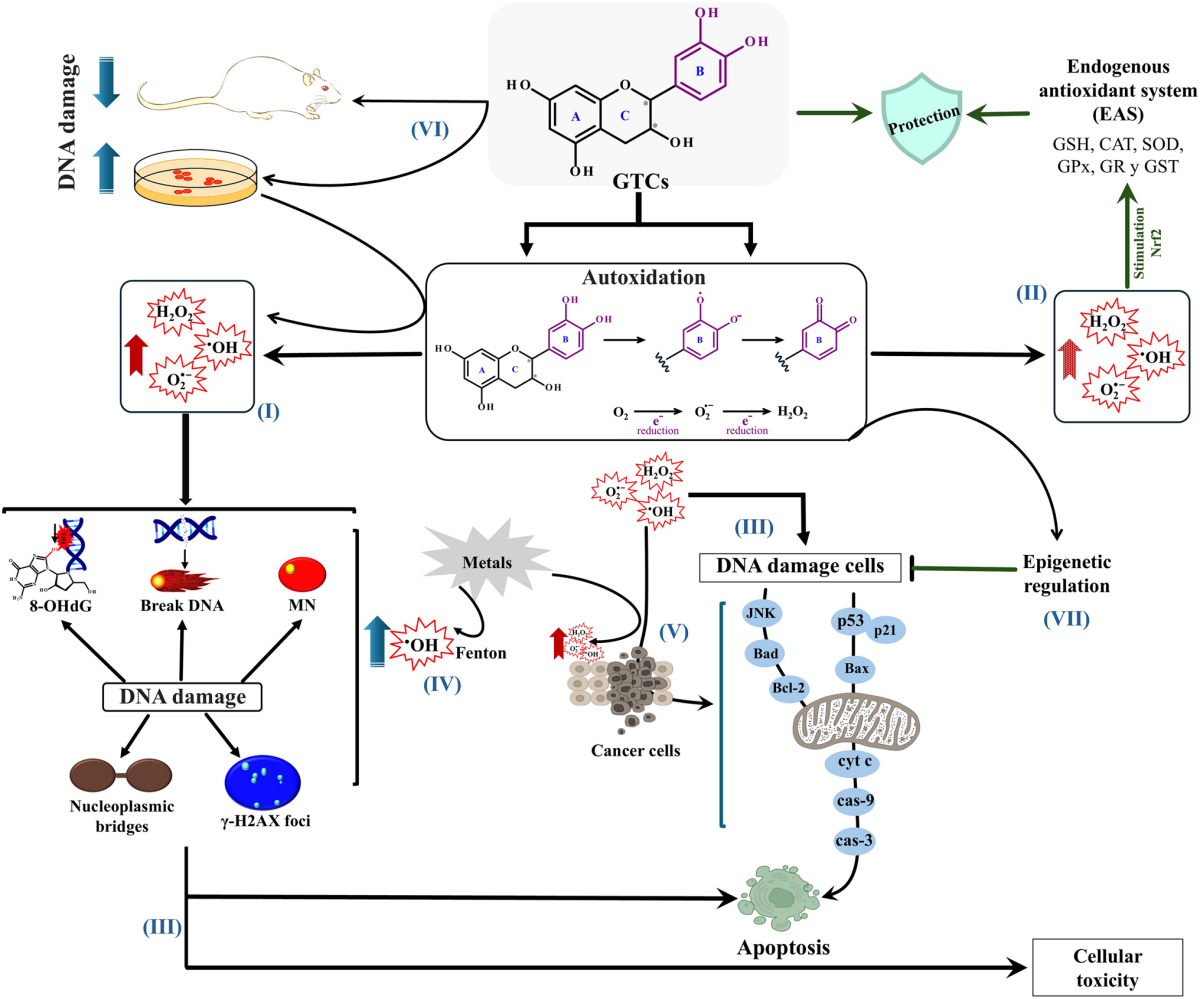
Mechanistic overview of green tea catechins’ genotoxic effects: (I) High ROS from auto-oxidation–elevated ROS levels generated during GTC autooxidation might interact with DNA, potentially inducing damage. (II) Low ROS activation of Nrf2–At lower ROS levels, GTCs activate Nrf2, reinforcing the antioxidant defense system and thereby contributing to antigenotoxic effects. (III) Apoptosis and cellular toxicity–Excessive ROS generated by high GTC doses or specific conditions might induce apoptosis and cellular toxicity, notably under certain environmental or experimental parameters. (IV) Metal presence–The presence of metals fosters enhanced ROS production through redox cycling, likely via Fenton-type reactions, leading to highly reactive •OH radicals that damage DNA. (V) In vitro* vs. *in vivo discrepancies–While GTCs may induce DNA damage in vitro, physiologic regulatory mechanisms in vivo help temper this effect, reducing genotoxic impact in living organisms

Although GTCs are primarily recognized for their antioxidant properties, several investigators suggest that under certain conditions, these compounds might exhibit prooxidant activities capable of inducing genotoxic effects (García-Rodríguez et al. 2022). This dual behavior arises from GTCs ability to both scavenge free radicals and generate ROS through autooxidation (Olson et al. 2020; Ouyang et al. 2020; Zhao et al. 2022). The ROS generated during autooxidation may directly interact with DNA, leading to damage (Fig. 3-I).

In vitro findings suggest that GTCs might increase number of DNA breaks (detected via comet assays), induce MN formation and γ-H2AX, and promote nucleoplasmic bridge formation in a concentration-dependent manner, with outcomes varying by cell type (Farhan et al. 2017; 2022; Ho et al. 2013; Hossain et al. 2014; Pereyra-Vergara et al. 2020; Prasad and Katiyar 2015; Udroiu et al. 2019). This DNA damage appears to result from ROS overproduction, as the use of selective ROS scavengers such as SOD, CAT or thiourea decrease the observed damage (Farhan et al. 2017). Ho et al. (2013) reported that higher green tea concentrations (0.05% w/v) generated significant H_2_O_2_ levels, leading to DNA damage, while lower concentrations (0.005% and 0.01% w/v) displayed genoprotective effects independent of CAT activity. These observations suggest that the tipping point between antigenotoxic and genotoxic roles of GTCs may rely upon H_2_O_2_ levels and related antioxidant responses. At lower ROS concentrations, Nrf2 upregulation (Fig. 3-II) might reinforce antioxidant defenses, whereas excessive ROS triggers apoptosis and cytotoxicity (Fig. 3-III).

In the presence of metals, GTCs’ prooxidant effects and subsequent DNA damage may intensify through redox cycling, likely via Fenton-type reactions that generate •OH (Fig. 3-IV). Furukawa et al. (2003) reported that EGCG induced oxidative DNA damage in bovine thymus DNA under oxidative stress conditions involving metal ions and H₂O₂, possibly through EGCG’s conversion to o-quinone and subsequent ROS production. Consequently, although GTCs induce genotoxic effects, these effects frequently coincide with apoptosis induction, aiding in the removal of genetically compromised cells. Farhan et al. (2022) found that C, EGCG, EGC, and EC inhibit cancer cell proliferation in a concentration-dependent manner, predominantly by stimulating apoptosis via elevated ROS levels. In this context, GTCs particularly EGCG display notable cytotoxic effects on tumor cells through mechanisms involving H_2_O_2_ generation mediated by EGCG’s pyrogallol moiety, which diminishes metal ions and sparks Fenton reactions that form ROS, including •OH (Kim et al. 2014). Catechins selectively target tumor cells by raising ROS levels above a threshold, thus preferentially triggering apoptosis. Malignant cells, often rich in copper (Cu), might further enhance ROS production via Fenton-like reactions, enhancing the selective cytotoxicity of catechins (Farhan et al. 2022). In agreement with these findings, Koňariková et al. (2015) and Durgo et al. (2011) showed that green tea extracts and EGCG induced DNA damage in human carcinoma cell lines including colon, breast and larynx, while not markedly affecting healthy cells. In breast cancer models, both EC and ECG promoted apoptosis by upregulating pro-apoptotic proteins Bad and Bax triggering cytochrome c (cyt c) release and subsequent caspase-3 (cas-3) activation (Farhan et al. 2017; Pereyra-Vergara et al. 2020). GTCs further inhibited the growth, proliferation, and metastasis of various cancer cells in in vitro and in vivo settings (Aggarwal et al. 2022; Farhan 2022). Notably, combining EGCG with a COX-2 inhibitor (e.g., NS-398) yielded synergistic anticancer effects by enhancing apoptosis. This synergy was evidenced by increased levels of pro-apoptotic markers (BAX, pro-caspase-6, pro-caspase-9), enhanced PARP cleavage, and suppressed NF-κB activity, accompanied by selective COX-2 inhibition in prostate cancer cell lines (LNCaP, PC-3) (Adhami et al. 2007). Further, Kciuk et al. (2023) noted in vivo that EGCG effectively inhibited early-stage prostate cancer by reducing cell proliferation and inducing apoptosis, although these effects appear to be diminished in later stages. Overall, these data indicate that GTC-induced oxidative stress might be designed to selectively target cancer cells via ROS-mediated apoptosis, facilitating the elimination of cells with significant genetic or cellular damage and reducing malignancy risk. In this respect, programmed cell death often driven by oxidative stress serves as a critical mechanism for eliminating genetically compromised cells once other protective measures fail, rendering GTCs’ genotoxic effects potentially advantageous for cancer prevention (Fig. 3-V). Further, these findings emphasize the multifaceted anticancer mechanisms of GTCs, demonstrating that these compounds might modulate apoptotic pathways and thereby create promising therapeutic opportunities in cancer treatment.

Although considerable research addresses GTCs’ antioxidant properties, fewer studies scrutinized their genotoxic potential. Some observed genotoxic outcomes might arise from experimental artifacts such as elevated ROS levels in certain cell-culture conditions (Hossain et al. 2014; Hou et al. 2005; Sinha et al. 2017; Udroiu et al. 2019). Although EGCG induces concentration-dependent DNA damage in human lymphocytes and Nalm6 cells, dietary doses rarely produce significant ROS formation or toxicity (Bertram et al. 2003). This discrepancy suggests that while GTCs may induce DNA damage in vitro, in vivo genotoxicity might be moderated by physiologic pathways managing oxidative stress and DNA repair (Fig. 3-VI).

Tables 1, 2 summarizes the biologic activities of GTCs, focusing on both antigenotoxic and genotoxic effects presented in this review. Each entry details the study model, primary metrics, outcomes, and modes of action including ROS scavenging, EAS regulation, DNA repair activation, and apoptosis induction. The dual properties of GTCs as antioxidants and prooxidants indicate their complex influence on antigenotoxic and genotoxic outcomes. It important to note that GTCs may safeguard or initiate DNA damage depending upon variables such as concentration, cellular environment, co-present metals, and experimental protocols. Clarifying these elements is key to refining the therapeutic applications of GTCs. Additional research is essential to define conditions that favor their beneficial antioxidant properties to counteract detrimental prooxidant effects, ultimately shaping safe and effective usage guidelines. By elucidating these mechanisms, investigators might better exploit GTCs’ beneficial health benefits while minimizing risks, culminating in more precise dosage recommendations in both clinical and preventive conditions.

**Table 2:** Overview of in vitro studies demonstrating genotoxic effects of green tea catechins

Treatment **(**Duration)	Study design	Experimental model	Key observations/findings	Bioactivities demonstrated and *target condition*	References
Green tea (0.005, 0.01, 0.05% w/v)	In vitro	Human lymphocytes	[0.005 and 0.01%] ⇩ DNA double-strand breaks [0.05%] ⇧ DNA double-strand breaks ⇧ H_2_O_2_	Antigenotoxic Genotoxic Prooxidant*—Human lymphocytes presence/absence CAT*	Ho et al. (2013)
Mixture of green tea polyphenols (10, 20, 40 y 60 μg/mL)	In vitro	A375, Hs294t, and SK-Mel28 human melanoma cells	⇧DNA breaks ⇩ HDAC ⇩ I HDAC ⇧ HAT ⇩ Cell proliferation in cancer cells	Genotoxic Anticarcinogenic*—Melanoma*	Prasad and Katiyar (2015)
EC (350 μM)	In vitro	MDA-M-B231 and MCF-7 breast cancer cells, normal breast epithelial cells	⇧DNA fragmentation ⇧ ROS ⇧ cyt c ⇧Bad and Bax ⇧ pro-cas-3 ⇧ Apoptotic cell ⇩ Cell proliferation	Genotoxic Apoptotic Antiproliferative Cytoprotective Prooxidant Anticarcinogenic*—Breast cancer*	Pereyra-Vergara et al. (2020)
ECG (10, 25, 50 μM)	In vitro	Human lymphocytes, breast cancer cells, normal breast epithelial cells	⇧DNA breaks ⇧ ROS ⇩ Cell proliferation in cancer cells	Genotoxic Prooxidant Antiproliferative Anticarcinogenic*—Breast cancer in presence of transition metal ions*	Farhan et al. (2017)
Catechin, EGCG, EGC, EC (10, 25, 50 mM)	In vitro	MCF-10A and cancer lines, PC-3, MDA-MB-231, BxPC-3, and MiaPaCa-2	⇧DNA breaks ⇧ ROS ⇧ Apoptotic cell ⇩ Cell proliferation in cancer cells	Genotoxic Prooxidant Antiproliferative Anticarcinogenic*—Cancer lines in presence of transition metal ions*	Farhan et al. (2022)
EGCG (10, 20 µg/mL)	In vitro	p53R and HeLa (ATCC)	⇧DNA breaks	Genotoxic Genoprotective adaptations*—p53R and HeLa in presence and absence of salivary α-amylase, serum albumin, and myoglobin*	Hossain et al. (2014)
EGCG (1, 5, 10 μg/mL)	In vitro	U251 human glioblastoma cells	⇧Micronuclei ⇧ γ-H2AX ⇩ Telomerase activity ⇩ % viable cells ⇧ Senescent cells	Genotoxic Cytotoxic Senescence*—Glioblastoma*	Udroiu et al. (2019)

⇩ Decrease or inhibition; ⇧ Increase

## Implications for health

GTCs have demonstrated considerable potential for beneficial health promotion and disease prevention. Notably, GTCs were reported to be effective in preventing excessive weight gain, alleviating metabolic syndrome symptoms, and reducing the risk of chronic illnesses such as diabetes and cardiovascular diseases (Cabrera et al. 2006; Samanta 2022; Yang et al. 2018a). GTCs also exhibit anti-aging, anti-atherosclerotic, and neuroprotective effects (Liu et al. 2023; Samanta 2022; Zhao et al. 2022). While the antigenotoxic properties of catechins are effective in lowering the risk of specific cancers (Cabrera et al. 2006; Farhan 2022), GTCs also display anti-inflammatory effects and decrease lipid peroxidation, key factors in the onset of various diseases, including cancer (Gu et al 2018; Chen et al. 2018; Zhao et al. 2018). Protective characteristics of GTCs largely derive from the potent antioxidant activity of catechins, which neutralize free radicals and protect against oxidative damage (Hajam et al. 2023). Further, GTCs demonstrate antimicrobial, antiviral, and antiallergenic activities (Hu et al. 2018; Samanta 2022).

Studies in animal models showed that GTCs inhibit tumorigenesis in various organs supported by over 20 investigations on lung cancer and more than 30 on digestive tract cancers through dietary green tea, polyphenol extracts, or EGCG supplementation (Yang et al. 2018a). Beyond their recognized antioxidant functions, GTCs exert anticancer effects via multiple mechanisms, including direct protein interactions, enzyme inhibition, and cell signaling modulation (Aggarwal et al. 2022; Farhan 2022). GTCs especially EGCG demonstrated a significant ability to regulate gene expression by binding to DNA and RNA in CpG-rich regions (Qadir et al. 2023). By modulating DNA methyltransferases (DNMT1, DNMT3a, DNMT3b), GTCs may reactivate tumor suppressor genes silenced by hypermethylation (Kim et al. 2014; Sur and Panda 2017; Udroiu et al. 2019). Evidence also indicates that green tea extracts modulate DNA methyltransferase activity and guard against DNA hypomethylation induced by carcinogens such as 7,12-dimethylbenz(a)anthracene (DMBA) (Szabo et al. 2021) as evidenced by blocking hypermethylation of tumor suppressor genes, which affect critical cellular pathways (Qadir et al. 2023), GTCs present promising therapeutic options for cancer prevention and treatment. Overall, these findings demonstrate the chemopreventive potential of GTCs particularly EGCG via their influence on epigenetic mechanisms (Figs. 2-VIII, 3-VII).

The antigenotoxic attributes of GTCs may yield benefits for chronic diseases and cancer. The anti-inflammatory effects help indirectly lower oxidative DNA damage by decreasing ROS production, a recognized outcome of inflammation. Investigators showed that GTC therapies lessens inflammation associated with myocardial injury, hepatic ischemia–reperfusion, Parkinson’s disease, and alcoholic liver disease (Chen et al. 2018; Liang et al. 2019; Liu et al. 2022; Tak et al. 2016; Tseng et al. 2020; Zhao et al. 2018). Further, GTCs were reported to protect against effects initiated by environmental toxins by alleviating inflammation and mutations triggered by perfluorodecanoic acid (PFDA), tetrabromobisphenol A (TBBPA), and fluoride in the lung and kidney (Lv et al. 2024; Shanmugam et al. 2020; Wang et al. 2020), and in conditions such as Parkinson’s and alcoholic liver diseases (Tseng et al. 2020; Zhao et al. 2018). In human ventricular cardiomyocytes, EGCG lowered ROS levels and enhanced SOD and CAT activities, thereby ameliorating smoke-induced inflammation and in animal model of Parkinson’s disease (Liang et al. 2019; Tseng et al. 2020). Across these studies, GTC treatment consistently diminished pro-inflammatory cytokines (IL-1β, IL-6, IL-8, TNF-α) while elevating anti-inflammatory IL-10, reducing oxidative stress, and promoting anti-apoptotic signaling (Table 3). Such outcomes feature enhanced Bcl-2 expression and decreased levels of pro-apoptotic proteins Bad, Bax, cas-3, cas-9 and cyt c, coupled with increased endogenous antioxidant defenses (Chen et al. 2018; Liang et al. 2019; Lv et al. 2024; Tak et al. 2016; Wang et al. 2020; Wongmekiat et al. 2018). In addition, Hamed et al (2025) found matcha treatments to inhibit the NF-κB/MAPK pathway, known to worsen inflammation and oxidative DNA damage, thus effectively mitigating radiation-induced inflammation.These findings suggest that the anti-inflammatory actions of GTCs may help mitigate chronic oxidative stress and, consequently, protect against oxidative DNA damage.

**Table 3:** Overview of in vivo and in vitro studies demonstrating the anti-inflammatory, antioxidant, and cytoprotective effects of green tea catechins

Treatment (Duration)	Study design	Experimental model	Key observations/findings	Bioactivities demonstrated and *target condition*	References
Fermented green tea 6, 12 mg/mL of tea extract crude liquor (5 days orally)	In vivo	Male C57BL/6N mice	⇩MDA ⇧ SOD and GPx ⇩ TNF-α, IL-1β, IL-6 ⇩ Inflammatory gene expression	Protection lipid peroxidation Antioxidant Anti-inflammatory Cytoprotective*—Cigarette smoke*	Liu et al. (2022)
EGCG 10 μM (30 min)	In vitro	Adult human ventricular cardiomyocytes (AC16)	⇧TAC ⇧ SOD and CAT ⇩ ROS ⇧ Bcl-2 ⇩ Bax ⇩ Apoptotic cells ⇩ IL-8	Antioxidant Anti-apoptotic Anti-inflammatory Cardioprotective*—Cigarette smoke*	Liang et al. (2019)
EGCG 5 mg/kg (28 days by ip)	In vivo	Male Sprague–Dawley rats	⇩MDA ⇧ GPx and SOD ⇩ CAT ⇧ Nrf-2 ⇧ HO-1, NOQ1 ⇩ ROS ⇩ cas-3 ⇩ Apoptotic cells	Protection lipid peroxidation Antioxidant Anti-apoptotic Protection bladder dysfunction*—Bladder dysfunction*	Gu et al. (2018)
EGCG 10 mg/kg (28 days by ip)	In vivo	Male C57/B6 mice	⇩MDA ⇧ SOD and GPx ⇩ TNF-α, IL-1β, IL-6 ⇩ Bax ⇩ cas-3	Protection lipid peroxidation Antioxidant Anti-inflammatory Anti-apoptotic Cardioprotective*—Myocardial injury*	Chen et al. (2018)
EGCG 50 mg/kg (Single by ip)	In vivo	Male C57BL/6 mice	⇩MDA ⇩ GSH:GSSG ⇩ GSH ⇧ HO-1 and TRXr-1 ⇩ TNF-α, IL-1β, IL-6 ⇧ IL-10 ⇧ Bcl-2 ⇩ Bax ⇩ cyt c ⇩ cas-3 and cas-9	Protection lipid peroxidation Antioxidant Anti-inflammatory Anti-apoptotic Hepatoprotective*—Hepatic ischemia–reperfusion*	Tak et al. (2016)
EGCG 100, 300 mg/kg (21 days by ip)	In vivo	Male Wistar rats	⇩TBARS ⇧ SOD and CAT ⇧ GSH ⇩ TNF-α, IL-1β, IL-6 ⇩ cas-3 ⇧ Neurotransmitter	Protection lipid peroxidation Antioxidant Anti-inflammatory Anti-apoptotic Neuroprotective*—Animal model of Parkinson’s disease*	Tseng et al. (2020)
EGCG 0,5% [≈0,3 mmol/kg] (35 days orally)	In vivo	Male ICR (CD-1) mice	⇩MDA ⇧ SOD, CAT and GPx ⇧ GSH ⇧ HO-1 ⇩ TNF-α, IL-6 ⇩ cas-3	Protection lipid peroxidation Antioxidant Anti-inflammatory Anti-apoptotic Hepatoprotective*—Alcoholic liver disease*	Zhao et al. (2018)
Green tea infusion 250 mg/kg (14 days orally)	In vivo	Male Swiss albino mice	⇩TBARS ⇩ Protein carbonylation ⇩ MDA ⇧ GPx and GST ⇩ DNA breaks	Protection lipid peroxidation Antioxidant Antigenotoxic Reproprotective*—Reproductive system* *(Cyclophosmamide)*	Zanchi et al. (2015)
Green tea extract 7.5 mg/kg (14 days by gavage) 30 μg/mL (12 h)	In vivo In vitro	Male c57 rats A549 cells	⇩MDA ⇧ T-SOD, CAT, GSH ⇩ ROS ⇩ TNF-α, IL-1β, IL-6 ⇧ IL-10 ⇩ cas-3, cas-7, cas-9, Bax ⇧ Bcl-2 ⇩ Apoptotic cells	Protection lipid peroxidation Antioxidant Anti-inflammatory Anti-apoptotic Cytoprotective*—Lung injury* *(TBBPA)*	Lv et al. (2024)
EGCG 40 mg/kg (28 days orally)	In vivo	Male Wistar albino rats	⇩MDA and MPO ⇧ SOD, CAT, GPx, GR and GST ⇧ GSH ⇧ α-TOH ⇧ Nrf-2 ⇧ HO-1 ⇩ ROS ⇩ TNF-α, IL-1β, IL-6	Protection lipid peroxidation Antioxidant Anti-inflammatory Protection lung oxidative damage*—Lung damage* *(Fluoride)*	Shanmugam et al. (2020)
P60-GT 25, 50, 100 mg/kg (7 days by ip)	In vivo	Male Kunming mice	⇩TBARS ⇧ GSH ⇩ ROS ⇩ ALT and AST ⇩ BAX ⇩ cas-3 ⇧ Viable cells ⇩ Micronuclei ⇩ Chromosomal aberrations	Protection lipid peroxidation Antioxidant Antigenotoxic Cytoprotective Hepatoprotective*—Liver damage* *(Patulin)*	Song et al. (2014)
Green tea polyphenols or EGCG 0.32% [w/v] (49 days orally)	In vivo	Male CD-1 (ICR) mice	⇩TBARS ⇩ MDA ⇧ SOD, CAT and GPx ⇩ ROS ⇩ TNF-α, IL-1β, IL-6, IL-8 ⇧ Bcl-2 ⇩ Bax ⇩ cas-3	Protection lipid peroxidation Antioxidant Anti-inflammatory Anti-apoptotic Hepatoprotective*—Liver damage* *(PFDA)*	Wang et al. (2020)
Matcha green tea 150 mg/kg (14 days orally)	In vivo	Female Wistar albino rats	⇩MDA ⇧ SOD and GST ⇩ NF-κB/MAPK signaling ⇩ DNA fragmentation	Protection lipid peroxidation Antioxidant Anti-inflammatory Antigenotoxic Hepatoprotective*—Liver damage* *(Ionizing radiation)*	Hamed et al. (2025)
Green tea extract 100 mg/kg (60 days orally)	In vivo	Male albino Sprague–Dawley rats	⇩MDA ⇩ NO ⇧ TAC ⇩ cyt c ⇩ Apoptotic cell ⇧ DNA content	Protection lipid peroxidation Antioxidant Anti-apoptotic Antigenotoxic Hepatoprotective*—Molecular fibrogenesis* *(Fe)*	Al-Basher (2019)
(+)-Catechin 25, 50, 100 mg/kg (28 days orally)	In vivo	Male Wistar albino rats	⇩MDA ⇧ Mitochondrial membrane potential ⇧ SOD and CAT ⇩ ROS ⇩ NO ⇩ TNF-α ⇩ Apoptotic cells	Protection lipid peroxidation Antioxidant Anti-inflammatory Anti-apoptotic Renoprotective*—Kidney damage* *(Cd)*	Wongmekiat et al. (2018)
Green tea extract 190, 380 mg/L (40 min)	In vitro	*Allium cepa L*	⇩MDA ⇩ SOD, CAT ⇩ ROS ⇩ Micronuclei ⇩ Chromosomal aberrations	Protection lipid peroxidation Antioxidant Antigenotoxic Cytoprotective*—Metal toxicity (Hg)*	Çavuşoğlu et al. (2022)
EGCG 25, 50 mg/kg) (15 days orally)	In vivo	Female balb/C mice	⇩TBARS ⇧ CAT, SOD, GST and GR ⇧ GSH ⇩ ROS ⇩ DNA fragmentation ⇩ % DNA and tail length ⇩ Micronuclei ⇩ Chromosomal aberrations	Protection lipid peroxidation Antioxidant Antigenotoxic Protection from oxidative DNA damage Hepatoprotective Renoprotective*—Metal toxicity (As)*	Kaushal et al. (2019)
EGCG 20, 50 μM (24 h)	In vitro	Human lymphocytes	⇩MDA ⇧ GSH ⇩ Micronuclei	Protection lipid peroxidation Antioxidant Antigenotoxic*—Malathion*	Taghavi et al. (2020)

⇩ Decrease or inhibition; ⇧ Increase

**Fig. 4:**
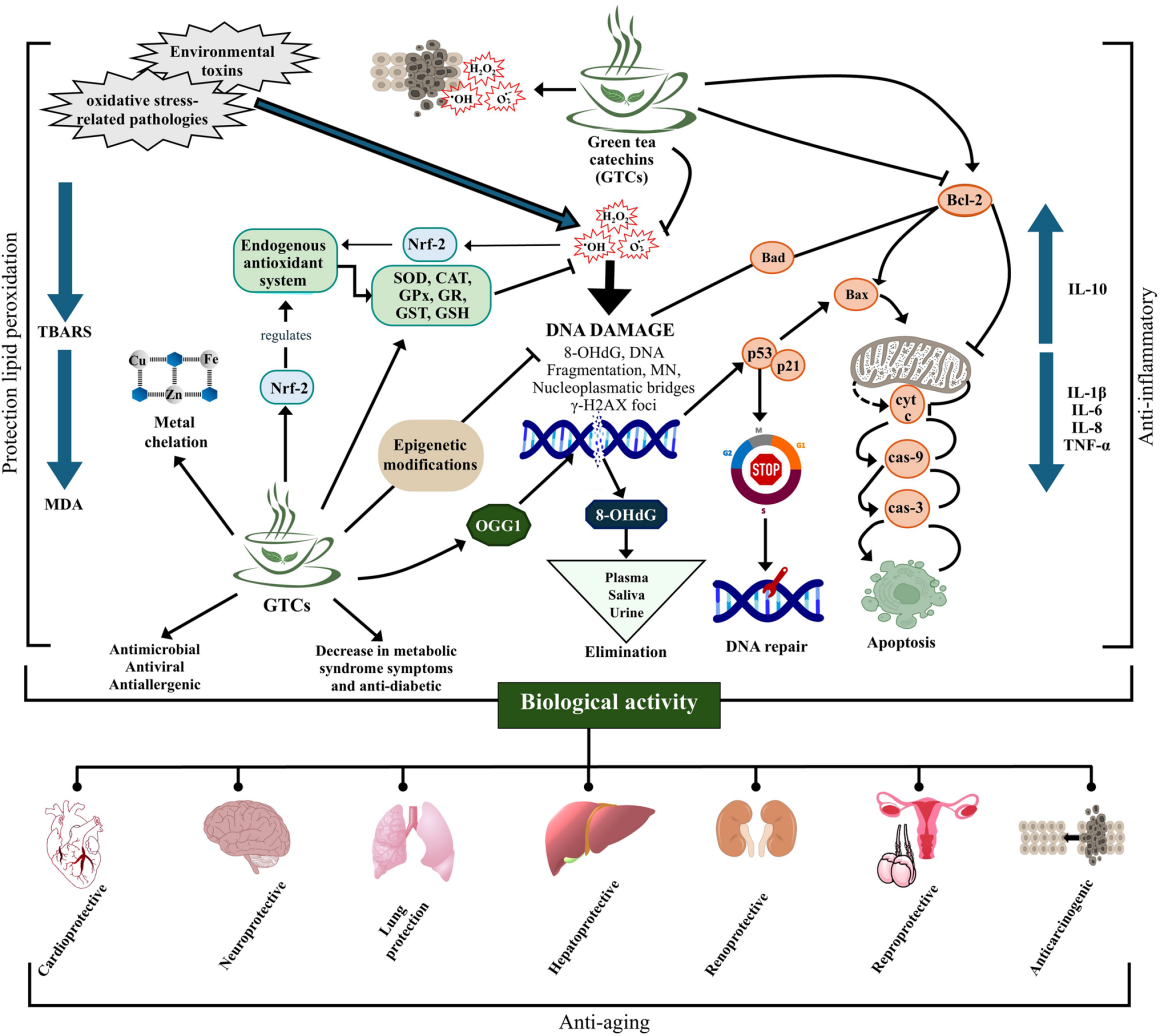
Comprehensive schematic of green tea catechins (GTCs) in oxidative stress mitigation, DNA damage prevention, and overall health benefits. Exposure to environmental toxins and oxidative stress-related pathologies may elevate ROS levels and DNA damage, indicated by lipid peroxidation markers (TBARS, MDA) and DNA damage endpoints (breaks, fragmentation, MN, nucleoplasmic bridges). GTCs counteract these effects by directly scavenging ROS or activating the Nrf2 pathway, which upregulates key antioxidant enzymes (SOD, CAT, GPx, GR, GST) and preserves GSH. This antioxidant response diminishes oxidative damage, modulates inflammatory cytokines (IL-10, IL-8, IL-1β, IL-6), and supports genomic stability by enhancing DNA repair mechanisms (e.g., OGG1) and affecting epigenetic regulation. In addition, GTCs regulate apoptosis by balancing pro- and anti-apoptotic factors (Bax, Bcl-2) and activating caspases (casp-3, casp-9), enabling either cell-cycle arrest for DNA repair or cell death when damage is irreparable. Under certain circumstances, however, GTCs may exhibit genotoxic effects—particularly in cancer cells—by promoting apoptosis. This dual behavior underlies the broad biologic benefits attributed to GTCs, including improvements in metabolic, hepatic, cardiovascular, neurologic, pulmonary, renal, and reproductive functions, as well as antimicrobial, antiviral, and antiallergenic properties. Collectively, these multifaceted actions contribute to overall health and, ultimately, anti-aging benefits

GTCs also inhibit lipid peroxidation, as evidenced by decreases in malondialdehyde (MDA) and thiobarbituric acid reactive substances (TBARS), byproducts that may adversely interact with proteins and DNA, leading to genotoxic damage and altered cellular signaling. By diminishing ROS levels and regulating or modulating EAS, GTCs alleviate oxidative stress and block lipid peroxidation, both integral to chronic disease and carcinogenesis (Sur and Panda 2017). Several investigators indicated that green tea infusions, extracts, or isolated catechins stimulated antioxidant enzymes GPx, GST and SOD after exposure to heavy metals, mutagens such as cyclophosphamide, patulin, or malathion, and radiation, reducing DNA damage and maintaining redox homeostasis (Al-Basher 2019; Çavuşoğlu et al. 2022; Hamed et al. 2025; Kaushal et al. 2019; Song et al. 2014; Taghavi et al. 2020; Zanchi et al. 2015). Data suggest that GTCs may help offset oxidative stress and protect cellular membranes by inhibiting lipid peroxidation. Figure 4 provides an overview of GTCs’ dual effects on cellular homeostasis, presenting the key mechanisms that drive GTCs health benefits.

### Bioavailability and therapeutic considerations

Epidemiologic evidence suggests that consuming at least four cups of green tea per day confers notable health benefits, potentially mitigating DNA damage and aiding in cancer prevention (Hu et al. 2018; Samanta 2022; Sur and Panda 2017). A standard 250-ml cup brewed from 2.5 g of tea leaves provides approximately 240–320 mg of catechins, with EGCG comprising 60–65% of this total (Gu et al. 2018; Shanmugam et al. 2020). However, the absorption and efficacy of GTCs depend upon several factors, including molecular structure, water solubility, chemical stability, food matrix interactions, co-consumed nutrients, and individual metabolic pathways (Aggarwal et al. 2022; Chow and Hakim 2011). Pharmacokinetic findings indicate that catechins reach peak plasma levels within 1.4 to 1.6 h post-ingestion; while EGCG primarily circulates in free form, EGC and EC typically undergo conjugation. Notably, EC, which contains fewer phenolic groups, exhibits higher bioavailability than the more structurally complex EGCG (Ouyang et al. 2020; Yang et al. 2018a, b). In addition, less than 1% of orally administered EGCG is detectable in the bloodstream, with an average plasma concentration of only 0.57 μM achieved after consuming 3 g of decaffeinated green tea. Consequently, some investigators recommend increasing systemic GTCs availability through higher doses, potentially up to 16 cups of green tea daily (Aggarwal et al. 2022; Chow and Hakim 2011). GTC stability is also pH-sensitive: acidic conditions (pH < 4) promote stability, whereas higher pH and temperature induce epimerization and modified bioactivity (Senanayake 2013).

While in vitro results do not always fully correlate to in vivo scenarios, in part due to limited bioavailability, physiologically relevant levels of GTCs were found to activate antioxidant defenses, bolster immune function, and lessen cellular damage under conditions of high oxidative stress (Aggarwal et al. 2022). Further, repeated exposure to GTCs may stimulate cellular adaptation, thereby promoting protective responses against potential genotoxic effects (Hossain et al. 2014; Liu et al. 2023). When GTCs interact with the gut microbiome, a critical factor in metabolism and bioavailability, these compounds may be converted into bioactive metabolites that may enhance their therapeutic benefits (Liu et al. 2023). Despite the significant insights into GTCs’ molecular mechanisms and therapeutic potential, their clinical translation of these compounds is challenged by bioavailability constraints and the multifactorial nature of disease. Additional pharmacokinetic and pharmacodynamic investigations are necessary to optimize the benefits of GTCs while minimizing potential adverse risks. Conclusive validation of GTCs’ antigenotoxic properties may further clarify their role in mitigating oxidative stress and DNA damage. Ultimately, more thorough investigations may pave the way for broader use of GTCs in clinical and public health arenas, once safe and effective dosing guidelines for preventive or adjunctive therapies are established.

## Conclusions and perspective

GTCs, particularly EGCG, hold significant potential for health promotion and disease prevention associated with oxidative stress and genetic damage. This review presented several key mechanisms underlying the antigenotoxic effects of GTCs, including: (1) antioxidant activity, which involved scavenging and inhibiting ROS to prevent oxidative damage; (2) regulation of the EAS as evidenced by enhancing the activity of detoxifying enzymes; (3) activation of DNA repair mechanisms including facilitation of removal of 8-OHdG adducts and other oxidative lesions; (4) induction of apoptosis by supporting the elimination of genetically compromised cells, particularly via prooxidant-induced apoptotic pathways; and (5) regulation of gene expression as evidenced by modulation of DNA and RNA interactions that influence transcription factors and epigenetic modifications. In addition, GTCs exhibited the capacity to chelate metal ions such as Cu, Fe, and Zn, thereby indirectly regulating transcription factors and enzymes, contributing to cellular homeostasis, and reducing inflammation.

The evidence reviewed indicates that GTCs may either protect against or induce DNA damage depending upon factors such as concentration, cellular environment, metal ion presence, and cell type. The genotoxic effects of GTCs are primarily linked to ROS generation through autooxidation, a dual behavior that presents intriguing therapeutic possibilities. Specifically, higher ROS concentrations might selectively target cancer cells through ROS-mediated apoptosis, while lower ROS levels generated during autooxidation may stimulate antioxidant defenses via Nrf2 activation.

Both in vitro and in vivo evidence demonstrates that GTCs may offer significant health benefits and aid in preventing DNA damage–related diseases by diminishing inflammation, inhibiting lipid peroxidation, and modulating immune responses. While GTCs’ protective role against oxidative stress and DNA damage is well documented, the prooxidant effects warrant cautious interpretation. Future research might to focus on (1) optimizing formulations to improve GTC bioavailability, (2) assessing long-term health outcomes associated with GTC consumption, (3) rigorously evaluating safety and potential side effects at higher doses, as well as therapeutic efficacy in specific diseases and stages, and (4) investigating gut microbiome interactions, given their influence on GTC metabolism and effectiveness. Overall, the multifaceted activities of GTCs emphasize their potential as both antigenotoxic and, under particular circumstances, genotoxic agents. This dual role constitutes a promising area for further research and clinical application in preventive and complementary medicine, enhancing sustainable health strategies associated with natural compounds usage.

## Data Availability

The data supporting the findings of this study are available from the corresponding author upon reasonable request.
